# The Pandemic and Constitutionalism

**DOI:** 10.1007/s42439-022-00069-2

**Published:** 2022-11-17

**Authors:** Gábor Halmai

**Affiliations:** Law Department, Comparative Constitutional Law, European University Institute, Florence, Italy

**Keywords:** Pandemic, Emergency power, Illiberalism, Autocracy, Constitutionalism

## Abstract

The paper discusses the reactions of different political and constitutional systems reactions to the pandemic and also the impact of COVID to populism, constitutionalism, and autocracy. Beyond the choice between economic and health considerations also applied in liberal democratic countries, which have lead either to “under-” or “overreaction” to the pandemic, certain illiberal regimes used the crisis situation as a pretext to strengthen the autocratic character of their systems. In some cases, this needed an “underreach,” like in Poland to insist on the presidential election, which has been important to entrench the power of the governing party’s incumbent, elsewhere “overreach,” like in Hungary, where an unlimited emergency power of government has been introduced after the very first cases of contagion. These autocratic “overreactions” have breached the formerly used authoritarian legalistic approaches by openly violating their own illiberal constitutions. New “conservative” theories on “common good constitutionalism” emerged to legitimize the necessity of authoritative rule by the executive power. The paper concludes that one possibility to overcome authoritarian populism and restore constitutionalism in crisis situation such as the COVID-19 pandemic would be to rely on the involvement of the well informed public, one that is capable to understand and assess the advice of the meritocratic elite. This kind of participation would also help build up a constitutional culture necessary to preserve the values of constitutionalism.

A recent report on public opinion, populism, and the pandemic using data collected from 27 countries and over 80,000 individuals during the 2020–2021 pandemic has found evidence a reversal of the rise of populism without an erosion of the support for core democratic beliefs and principles, including less liberal attitudes with respect to basic civil rights and liberties and weaker preference for democratic government on the other (Foa et al. 2022). Is this good or a bad news regarding the state of constitutionalism in the times of a global pandemic? In this paper, I try to argue that it depends on what we think about populism and its relationship to constitutionalism.

## Emergency Measures and Constitutionalism

No one reasonably disputes that emergency situations like the coronavirus pandemic require special legal and constitutional measures even in fool-fledged constitutional democracies.^1^ These measures have to take into account various aspects, among them health and economic considerations, which can lead to different balancing outcomes between certain legitimate public interests, like public health, public order, security, and fundamental rights, such as the right to human dignity, right to life, freedom of movement, right to education, freedom of information and expression, and privacy. Even decisions of democratic legislators and governments potentially reviewed by independent judicial bodies can lead either to “under-” or “overreaction” to the pandemic.

As Francis Fukuyama has argued, it is not a matter of regime type why some countries have done better than others in dealing with the crisis so far (Fukuyama 2020). Some democracies have performed well, but others have not, and the same is true for autocracies. Therefore, for him, the factors responsible for successful pandemic responses have been state capacity, social trust, and leadership. Indeed, one can think of the old constitutional democracies, such as the US and the UK, which did not perform well due to lack of state capacity and/or effective leadership. On the one hand, all the states he mentions, which have used the crisis through overreaction to give themselves emergency powers, moving them still further away from democracy happen to be non-democracies. Tom Ginsburg and Mila Versteeg, after surveying 106 countries in the world where the executive is bound by judicial, legislative, or supranational oversight over their pandemic responses, came to the conclusion that in no fewer than 82% of the countries in their data at least one of these checks and balances could be observed.^2^ They have found only six democratic countries out of the surveyed 106 where no oversight could be detected: Australia, Botswana, Jamaica, Switzerland, Peru, and Guyana. Of course, this does not necessarily indicate that the executive’s reactions have been problematic, and conversely the formal oversight by non-independent bodies does not exclude the misuse of executive power in non-democratic regimes. This is because the lack of independence makes the executive power not bound by the oversight bodies. This is why the unbound character of the executive power cannot be equally assessed in democratic and autocratic systems.^3^ The overall positive picture of the Ginsburg-Versteeg survey has not been confirmed by the mentioned recent Cambridge report (Foa et al. 2022) or by earlier reports of Freedom House, which found that since the start of the pandemic, the state of democracy and human right, which tells a lot about the unbound character of the executive, has worsened in at least 80 countries out of the 192 nations surveyed by them.^4^

Besides the lack of oversight authoritarian regimes in Bangladesh, Belarus, Cambodia, China, Egypt, El Salvador, Syria, Thailand, Turkey, Uganda, Venezuela, and Vietnam have all detained critics, health workers, journalists, and opposition members, and implemented strong tools against the pandemic, including harsh censorship and even criminal sanctions against social media posts related to COVID-19 as an excuse to grab more power.^5^ Competent goverments that performed well in containing the virus can be considered stable democracies, such as Australia, New Zealand, Germany, Japan, or Taiwan (Diamond 2020). Some democracies have managed to adapt faster. For instance, in South Korea, the disease was being tamed by extensive tracing and widespread survaillance of possible carriers because the regime had recent experience to draw on its handling of the Mers outbrake of 2015, which also shaped the collective memory of the citizens. But it is true that COVID-19 infected the world with an ultimate uncertainty in a state in which democracy was already under threat. As David Runciman argues, the distinction between democracies and authoritarian regimes has been blurred: “under the lockdown, democracies reveal what they have in common with other political regimes: here too politics is ultimately about power and order” (Runciman 2020).

One of Tom Daly’s four categories of governments’ dealing with COVID-19 is reserved for “autocratic opportunists,” such as the Hungarian government, the “fantasists,” who denied the scientific facts of the pandemic consists autocracies, like China, illiberal states, such as Brazil, as well as traditional democracies, like the US, with huge leadership problems (Daly 2020). His two remaining categories of countries are rather considered democracies: the “effective rationalists,” like New Zealand, and the “constrained rationalists,” such as South Africa, whose constraint has been their limited state capacity.

Given the importance of strong state capacity and action to slow the pandemic, it will be hard to argue against a stronger state involvement during a national emergency. Also, according to Krastev and Leonard (2020), the virus strengthened rather than weakened national sovereignty. Interestingly enough, the contestation of the state’s “pandemic power’ has come from groups and movements spanning far-right populists, radical ecologists, wellness fanatics, and left-populist united by claims to “personal sovereignty,” “bodily autonomy,” and “bodily rights” (Bialasiewicz and Muehlenhoff 2020). Even the Dutch Prime Minsiter Mark Rutte described the Netherlands as a “grown-up country,” which did not need to be treated like children “to behave responsibly,” unlike other European citizenries (Bialasiewicz, 2020). But despite these rather minority views, most governments have assumed executive powers considered to be broadly necessary to contain the health crisis, and it remains uncertain whether these will entail long-term restrictions on democratic rights.^6^ Ivan Krastev calls it one of the COVID19-paradoxes: when people realize the threat to fundamental rights, they are rather inclined to reject authoritarian rule (Krastev 2020). Similarly, a recent study has shown that one-and-a-half year after the onset of the pandemic (mid-2021), there is no downstream effect of the fears and anxieties felt during the peak of COVID-19 on citizens’ support for fundamental elements of liberal democracy (Anghel and Schulte-Cloos 2022).

In the case of Belarus, one of the reasons of the “revolution of dignity”,^7^ which started in the Summer of 2020, was that the people realized that the absolute power did not provide security against COVID-19 but rather threatened their life.

The “semi-liberal”^8^ constitutional system of Israel has an in-built “overreach” element because a state of emergency existed already before the COVID-19 crisis: the Basic Law—the Government states that the Knesset “may, of its own initiative or, pursuant to a Government proposal, declare that a state of emergency exists.” This state of emergency was declared upon the establishment of the state of Israel and has been extended yearly ever since. Using the pandemic crisis, the government sought to employ surveillance technology to track those who tested positive to coronavirus. But as opposed to more authoritarian illiberal systems, in Israel the Supreme Court blocked the measures, disproportionately limiting fundamental rights.^9^

In illiberal regimes, all reactions can be motivated by the rulers’ authoritarian pursuits; in other words, they can use the crisis situation as a pretext to strengthen the autocratic character of their systems. In some cases, this needed an “underreach,” like the extreme ignorance of the virus by President Bolsenaro of Brazil, or in the US, where President Trump (after his first failed claim to have “absolute power”) responded insufficiently and incompetently with “executive underreach”^10^ to the pandemic. This meant that the US appallingly lacked emergency measures deployed at federal level. Similarly, the illiberal Polish government insisted on the presidential election, which was important to entrench the power of the governing party’s incumbent, despite the health risks.^11^

In far more illiberal states, an “overreach” has served the purpose of power grab, like in India, where the Modi government used the pandemic chaos to supress nationwide protests against the efforst by the Hindu Nationalist ruling party to marginalize religious minorities, which had been sustained 2 months before the coronavirus outbreak, such as the peaceful sit-in spearheaded largely by Muslim women in the Shaheen Bagh section of Delhi.^12^ Also, new restricting foreign NGOs from giving money to Indian ones, which could dismantle civil society organization, provide check on the increasingly authoritarian government (Kazmin 2020).

## Hungary as a Model Case: from “Illiberal Democracy” to Autocracy

Hungary represents a special case.^13^ Since the landslide victory of Viktor Orbán’s Fidesz party in the 2010 parliamentary election, the country according to Prime Minister Orbán’s self-definition became an “illiberal democracy,”^14^ with a new constitution motivated by the 2008–2009 financial crisis, and enacted with the exclusive votes of the governing party. In 2015, at the height of the migration crisis, the Parliament gave the power to the government to declare a “state of migration emergency” which allowed to hunt down and detain asylum seekers, punish those who assisted them, and use draconian new standards for rejecting asylum claims. The 2015 emergency law included sunset conditions that should have ended the emergency when the flow of refugees stopped. But nearly 5 years later with hardly a new refugee in sight, the government has renewed these emergency powers continuously through to the present day. Since both the 2014 and the 2018 parliamentary election results which renewed the two-third majority of Fidesz have been manipulated, some scholars started to question even the formal democratic character of the regime.^15^

Hungary was hit by COVID-19 in such a state of affairs. The government introduced an unlimited emergency power right after the very first cases of contagion. To make matters worse, the legal presumption on which both the initial emergency decree 40/2020 and the subsequent emergency statute (the Enabling Act) rest violated even Fidesz’ own illiberal constitution, the Fundamental Law of 2011, because it did not provide constitutional authorization either for the decree or for the Enabling Act. The Enabling Act was not even needed to cope with the crisis since the existing ordinary laws would have provided ample powers for dealing with a pandemic. The Parliament again unconstitutionally authorized the government to define the end of the pandemic and limited its own power to control the government’s decree power.^16^ There was also no chance that the packed Constitutional Court would have questioned the constitutionality of the emergency measures, which discredited local self-governments and opposition parties, limited freedom of expression, data protection, freedom of information, and labor rights.^17^

Although in mid-June 2020 the Hungarian Parliament repealed the law that gave outsized powers to the government, allowing it to override laws by decree with no time limit, the Hungarian government then faced EU budgetary sanctions so it retreated. Yet on the same day, when the parliament repealed the law that gave the government those extraordinary powers, the same parliament passed another law that gave the government the same powers with even fewer constraints.^18^

Even a less destructive nature of COVID-19 in Hungary provided a pretext for the Orbán government to dismantle the remnants of democratic character of its already “illiberal” state. In this rather autocratic state, the governmental government ssems to have the power to suspend also parliamentary elections, or change their rules at will as long as the emergency lasts. And when there are no regular elections or when their result is not uncertain, it has indeed nothing to do with democracy, as “institutionalized uncertainty,” a political system in which parties can lose elections.^19^ Together with losing its democratic character, the system, while violating its own constitution, made huge rhetoric efforts to keep the image of its “authoritarian legalist”^20^ character.

Assessing the reactions of the Hungarian government to the challanges of the pandemic the European People’s Party President and former Polish Prime Minister Donald Tusk was right when he claimed that Adolf Hitler’s jurist and prominent Nazi legal scholar Carl Schmitt would be proud of Hungarian Prime Minister Viktor Orbán.^21^ Carl Schmitt famously defended Hitler’s emergency measures by saying: the Führer protects the law. And that situation has been introduced by Viktor Orbán’s *Ermächtigungsgesetz* (Halmai 2020). This does not mean a completely lawless siutation, the constitution and laws are still in force, as the Weimar Constitution was not formally abolished during the Nazi era.^22^

## Conclusion: Populism, Constitutionalism, and Autocracy

As it has been shown, beyond the choice between economic and health considerations also applied in liberal democratic countries, which have lead either to “under-” or “overreaction” to the pandemic, certain illiberal regimes used the crisis situation as a pretext to strengthen the autocratic character of their systems. In some cases, this justified an “underreach,” like in Poland, in order to insist on the presidential election taking place, which was important to entrench the power of the incumbent governing party and despite the health risks involved. Elsewhere an “overreach” has served the same purpose, like in Hungary, where an unlimited emergency power of the government was introduced after the very first cases of contagion. Based on Carl Schmitt’s critique of liberal democracy, new theories on the “unbound executive” or “common good constitutionalism”^23^ emerged to legitimize the necessity of autoritative rule by (wannabe) autocrats.

The measures of illiberal governments as pretexts for further power grab demonstrated that liberalism in these cases is not besieged by democracy running amok (Luban 2020), or by populism for that matter, but rather by poor authoritarianism. Hence, neither the concept of “illiberal democracy” nor that of populism can serve as an analytical tool in order to understand the motivations of those would-be autocrats using COVID-19 to further their own interests.

Let me start with “illiberal democracy,” which is an oxymoron in my view, because there is no democracy without liberalism, and there also cannot be liberal rights without democracy.^24^ Those who perceive democracy as liberal by definition also claim that illiberalism is inherently hostile to the values associated with constitutionalism as an institutional aspect of liberal democracy: separation of powers, constraints on the will of the majority, human rights, and protections of minorities. Therefore, the similarly oxymonoric “illiberal” constitutionalism is necessarily authoritarian in character.^25^ The relationship between populism and constitutionalism is more complex due to the varieties of populism. For instance, Mark Tushnet and Bojan Bugaric in their recent book attempt to reconcile the relationship between certain types of populism with certain definition of constitutionalism (Tushnet and Bugaric 2021).In the barebones definition of populism and constitutionalism, there is no general conflict between the two. But populism can take a variety of forms with different political consequences due to the fact that it coexists with a variety of different host ideologies (Mudde 2004), which significantly determine how populism affects democracy and constitutionalism. This means that populism can have both negative and positive consequences for both. While some versions of populism can be consistent with (some versions of) constitutionalism,^26^ others cannot, and both right- and left-wing authoritarians can masquerade as populists, as the case of the current Hungarian and Polish government demonstrate. Resolving questions about possible conflicts always requires paying attention to actual local circumstances as in the case of India, Israel, or Brexit. One of the discussed positive impacts is the American anti-oligarchy concept of constitutionalism. While in his previous works Mark Tushnet expressed more understanding toward certain forms of authoritarian constitutionalism (Tushnet 2015), here it is the populism of the current Hungarian and Polish government that threatens the principles of constitutionalism. As I have argued elsewhere, the “authoritarian populist constitutionalism” is mostly a rhetoric, and not a real populist appeal to the “people” (Halmai 2019).

The COVID-19 pandemic has made the populist autocrats show their authoritarianism more openly.^27^ But using an undifferentiated concept of populism does not help understand all governmental reactions to COVID-19 either.^28^ For instance, the Italian government led by the populist Five Star Movement (after a terrible start due mostly to the regions of Lombardy and Veneto led by the far-right League) has been more or less successful in crushing the curve and containing the pandemic.^29^ Not all populists are autocrats abhoring restrains on the political executive.^30^ Moreover, economic populism can be justified against discretionary monetary policy or preferential treatment of foreign investors without democratic deliberation by both national and transnational executives (Rodrik 2018). For the same reason, blaming the people cannot help to understand the crisis of democracy also deepened by COVID-19.^31^

In his book, Michael Sandel (Sandel 2020) argues that populism is a reaction to the liberal left’s pursuit of meritocracy. In an interview, he claims the only way out of the crisis to dismantle the meritocratic assumptions that have morally rubber-stamped a society of winners and losers. Referring to the COVID-19 pandemic, he believes that the new appreciation of the value of supposedly unskilled, low-paid work offers a starting point for renewal: “This is a moment to begin a debate about the dignity of work; about the rewards of work both in terms of pay but also in terms of esteem. We now realise how deeply dependent we are, not just on doctors and nurses, but delivery workers, grocery store clerks, warehouse workers, lorry drivers, home healthcare providers and childcare workers, many of them in the gig economy. We call them key workers and yet these are oftentimes not the best paid or the most honoured workers.”^32^

It is also misleading to distinguish between antidemocrats, nativists, and populists, as the main challengers of political liberalism and liberal democracy (Pappas 2016). The illiberals are all antidemocrats, who delegitimize representative democracy’s normative foundation, and nativists, who protect the interests of the native-born or established inhabitants against those of immigrants, and they are populists, referring to the “pure people” against the “corrupt elite,” or during the pandemic crisis against experts, like virologists.^33^ As Roger Brubaker convincingly argued in 2017, several independent crisis, the financial crisis, the refugee crisis, the security crisis caused by terror attacks, and lately the crisis of public knowledge of fake news, alternative facts—and today we can add the coronavirus crisis—have converged and created a “perfect storm” conducive to populism (Brubaker 2017). But autocrats’ populism is “false”^34^ and they only use populist rhetoric, which does not necessarily correspond with these populists’ practice.^35^ In other words, we can answer the question of whether it is the groundswell of popular discontent in Europe and the Americas that is really threatening democracy^36^ in the negative. Paraphrasing James Carville’s *bon mot*, we should say “It’s the authoritarianism, stupid! The ultimate question is whether after this pandemic and many crises before it we can hope for the restoration of capitalism and democracy with it, or whether we have to face ‘the crisis of the crisis of capitalism,’”^37^ which will kill democracy as well.

One possibility to overcome authoritarian populism and restore constitutionalism in crisis situation such as the COVID-19 pandemic would be to rely on the the involvement of the well-informed public, one that is capable to understand and assess the advice of the meritocratic elite. This kind of participation would also help build up a constitutional culture necessary to preserve the values of constitutionalism.

## Footnotes

1 Among the very few exceptions are Slavoj Zizek, Giorgio Agamben and Peter Sloterdijk who rather warned of the dangers of any emergency power even in stable liberal democracies (see Joffe 2020). Agamben in his fear of every kind of emergency power went as far as calling the coronavirus as a’supposed pandemic’ and suggesting that emergency measures were’absolutely unwarranted.’ See Berg 2020.

2 In 52% of the countries (68% of democratic and 30% of autocratic ones), the legislatures, in 41% of them the judiciary (in 55% of the democracies, compared with 27% of autocracies), and in 34% (in 40% of democracies as compared with 28% of autocracies) subnational bodies have become involved in the decisions of the executive. See Ginsburg and Versteeg 2021.

3 Another comparative constitutional study comparing four European liberal democracies’ (Gemany, France, the UK, and Italy) efficiency in tackling the pandemic comes to the conclusion that the main divide among them is not between bound or unbound executives, between Madisonian or Schmittian models, but rather whether the “compulsion to legality” triggers virtuous or vacuous circles. See Golia et al. (2020). But how can this comparision possibly prove that the unbound character of the executive does not play any role in the efficiency of the reaction if none of the four selected countries has ever implemented the Schmittian model.

4 Democracy Under Lockdown. The Impact of COVID-9 on the Global Struggle for Freedom, Special Report 2020. Freedom House, https://freedomhouse.org/report/special-report/2020/democracy-under-lockdown?utm_campaign=wp_todays_worldview&utm_medium=email&utm_source=newsletter&wpisrc=nl_todayworld

5 See an overview of these countries emergency measures “Would-be Autocrats Are Using COVID-19 as an Excuse to Grab more Power,” The Ecomomist, April 23, 2020. https://www.economist.com/international/2020/04/23/would-be-autocrats-are-using-covid-19-as-an-excuse-to-grab-more-power.

6 Cf. a report of Carnegie Europe. https://carnegieeurope.eu/2020/06/23/how-coronavirus-tests-european-democracy-pub-82109.

7 See this phrase used by Adam Michnik, see Sierakowski (2020). https://www.project-syndicate.org/onpoint/belarus-revolution-of-dignity-by-adam-michnik-and-slawomir-sierakowski-2020-08.

8 Gila Stopler defines the state of the current Israeli constitutional system as “semi-liberal constitutionalism” (Stopler 2017).

9 See a detailed report in Brandes (2020).

10 See the term used by Gould and Pozen (2020). Kim Lane Scheppele uses the term “underreaction” to describe the same phenomenon, Scheppele (2020). See also the joint study of Pozen and Scheppele (2020).

11 See Drinóczi and Bien-Kacala (2020).

12 https://theconversation.com/coronavirus-versus-democracy-5-countries-where-emergency-powers-risk-abuse-135278.

13 The previously quoted Ginsburg-Versteeg survey also distinguishes Hungary where measures violated all three rule of law principles (temporally limited responses, judicial and legislative oversight, non-discriminatory application) reviewed in the 106 countries surveyed. See Ginsburg and Versteeg (2021).

14 Based on Fareed Zakaria’s use of term “illiberal democracy” (Zakaria 2003), it was PM Orbán of Hungary who first characterized his regime as such in his speech at the 28th Bálványos Summer Open University and Student Camp, 28 July 2018. Tusnádfürdő (Băile Tuşnad). http://www.miniszterelnok.hu/prime-minister-viktor-orbans-speech-at-the-29th-balvanyos-summer-open-university-and-student-camp/.

15 Larry Diamond for instance called the Hungarian system as “pseudo-democracy” (Diamond 2019). Similarly, Steven Levitsky and Lucan Way argued that “Orbán’s Hungary is a prime example of a competitive autocracy with an uneven playing field” (Levitsky and Way 2019). See also Bozóki and Hegedűs (2020), 1173. In 2015, Hungary was also reclassified from a consolidated democracy to a semi-consolidated democracy by Freedom House’s Nations in Transit report. https://freedomhouse.org/sites/default/files/2020-02/FH_NIT2015_06.06.15_FINAL.pdf. In its 2020 report, Freedom House went further declaring that Hungary has sunk from the the status of a semi-consolidated status to that of a hybrid regime of democracy and authoritarianism. https://freedomhouse.org/country/hungary/freedom-world/2020.

16 The Italian government did exactly the opposite: without having such rule in the constitution, it adopted a new decree law that included important safeguards, such as an obligation to report to parliament every two weeks.

17 About the details of the emergency measures of the Hungarian government, see Halmai and Scheppele (2020a, b), and Halmai and Scheppele (2020a).

18 See a detailed analysis of the new emergency power written by Halmai et al. (2020).

19 See this epigrammatic definition of democracy in Przeworski (1986), 58.

20 See the term used by Scheppele (2018).

21 https://rmx.news/article/article/hungarian-government-outraged-after-donald-tusk-claims-prominent-nazi-would-be-proud-of-pm-orban.

22 See Jiri Priban’s interview after the start of the Corona crisis: https://www.e15.cz/rozhovory/obdobi-prosperity-ceske-spolecnosti-prave-konci-mini-sociolog-jiri-priban-1369589

23 See for instance Harvard law professor Andrian Vermuele’s article published the day after Viktor Orbán’s Parliament enacted his version of the Enabling Act (Vermeule 2020).

24 Habermas (1994), 83–94. The English version see Habermas (1995). Also Juan José Linz and Alfred Stepan assert that if governments, even being freely elected violate the right of individuals and minorities, their regimes are not democracies. See Linz and Stepan (1996), 15. Similarly, János Kis claims that there is no such thing as nonliberal democracy, or non-democratic liberalism. See Kis (2019), 45–74. Those critics who argue that liberalism as a three hundreds year old concept predates liberal democracy forget that not only democracy but also liberalism presupposes general and equal suffrage.

25 Following Juan José Linz’s classical categories, authoritarianism is in between democratic and totalitarian political system (Linz 2000). Writing about Franco’s Spain Linz has already outlined the following four main charactersitics of authoritarianism: limited, not responsible political pluralism, without elaborate and guiding ideology, without political mobilization, and with formally ill-defined, but quite predictable limits of power (Linz 1970). About the constitutional markers of authoritarianism as a pretence of democracy, such as the lack of procedural rights, institutional guarantees, and public discourse see Tóth 2018.

26 For instance, Paul Blokker considers “populist constitutionalism” as an alternative, conservative understanding of constitutional order, which is, among other things, a reaction to injustices resulting from liberal democratic politics, to unbalanced emphasis on formalistic liberal institutions, rights, and norms, and an aggressive institutionalisation of a liberal understanding of law in the post-1989 transformation. See Blokker (2018) as well as Blokker (2019).

27 Similarly, Müller (2020).

28 See such a view in Weale (2020).

29 Paul Krugman compared the Italian success in weathering the coronavirus with the failure of the US to do the same. See Krugman (2020).

30 Among the many critics of anti-populism, see the book of Frank (2020), which praises the “real” populism of the American People’s Party in the 1890s, as well as FDR’s New Deal in the 1930s. He argues that the “empty culture-war fight” of the GOP and Trump (or Fidesz and Orbán in Hungary for that matter) is “false populism.” See an interview with him: MacDougald (2020).

31 See Eric Posner’s book (Posner 2020), which blames the American people for for Trump’s rise, criticized by Yale Law School historian, Samuel Moyn (Moyn 2020). Similarly, Joseph Weiler blamed the Hungarian people for supporting Orbán (Weiler 2020). See a critique by Kazai (2020).

32 https://www.theguardian.com/books/2020/sep/06/michael-sandel-the-populist-backlash-has-been-a-revolt-against-the-tyranny-of-merit?CMP=Share_iOSApp_Other.

33 While in my view nowadays all illiberals are populists, not all populists are necessarily illiberals; for instance, some of the left populists are not. Even in East-Central Europe the populism of the former Czech Prime Minister, Andrej Babis was lacking strong illiberal components. Contrary to my understanding, Andrew Arato and Jean Cohen, in analyzing the normative theory of left populism by Ernesto Laclau and Chantal Mouffe, respectively claim that left populism also cannot avoid illiberal authoritarianism inherent in the strategy and logic of populism despite the inclusionary and democratizing projects of the left movements it attaches to and despite the democratic socialist rhetoric of left populist leaders and their organic intellectuals. See Arato and Cohen (2022). In my view, it is certainly true for Latin American populist from Peron through Morales, Correa to Chavez, and Maduro, but not necessarily for European left populist parties, such as Podemos, the Five Star Movement, and Syriza. The last two did not even show serious illiberal pursuits while being in power.

34 The term “false” populism was used by Isaiah Berlin defining “the employment of populist ideas for undemocratic ends.” See Berlin (1968), 10.

35 About the use of populist rhetoric by Viktor Orbán and his government, see a more detailed description in Halmai (2019). Similarly, Gáspár Miklós Tamás argues that the Orbán government is “an ingenous old-new from of flexible and non-murderous dictatorship, but why is it ‘populist’?” (Tamás 2017). Based on Hannah Arendt republican conception, Camila Vergaga also distingushes populism from proto-totalitarianism, arguing that populism aims to increase the welfare of the plebeian masses, and therefore its relation to liberal democracy appears to be one of reform, while totalitarian movements, even when they use populist rhetoric want to destroy liberal democracy and constitutionalism altogether. See Vergara (2020).

36 Steven Hahn, ‘The Populist Specter. Is the Groundswell of Popular Discontent in Europe and the Americas What’s really Threatening Democracy?,” The Nation, January 28-February 4, 2019. https://www.thenation.com/article/archive/mounk-galston-deneen-eichengreen-the-populist-specter/.

37 This is the main claim of the recent book by Azmanova (Azmanova 2020).
